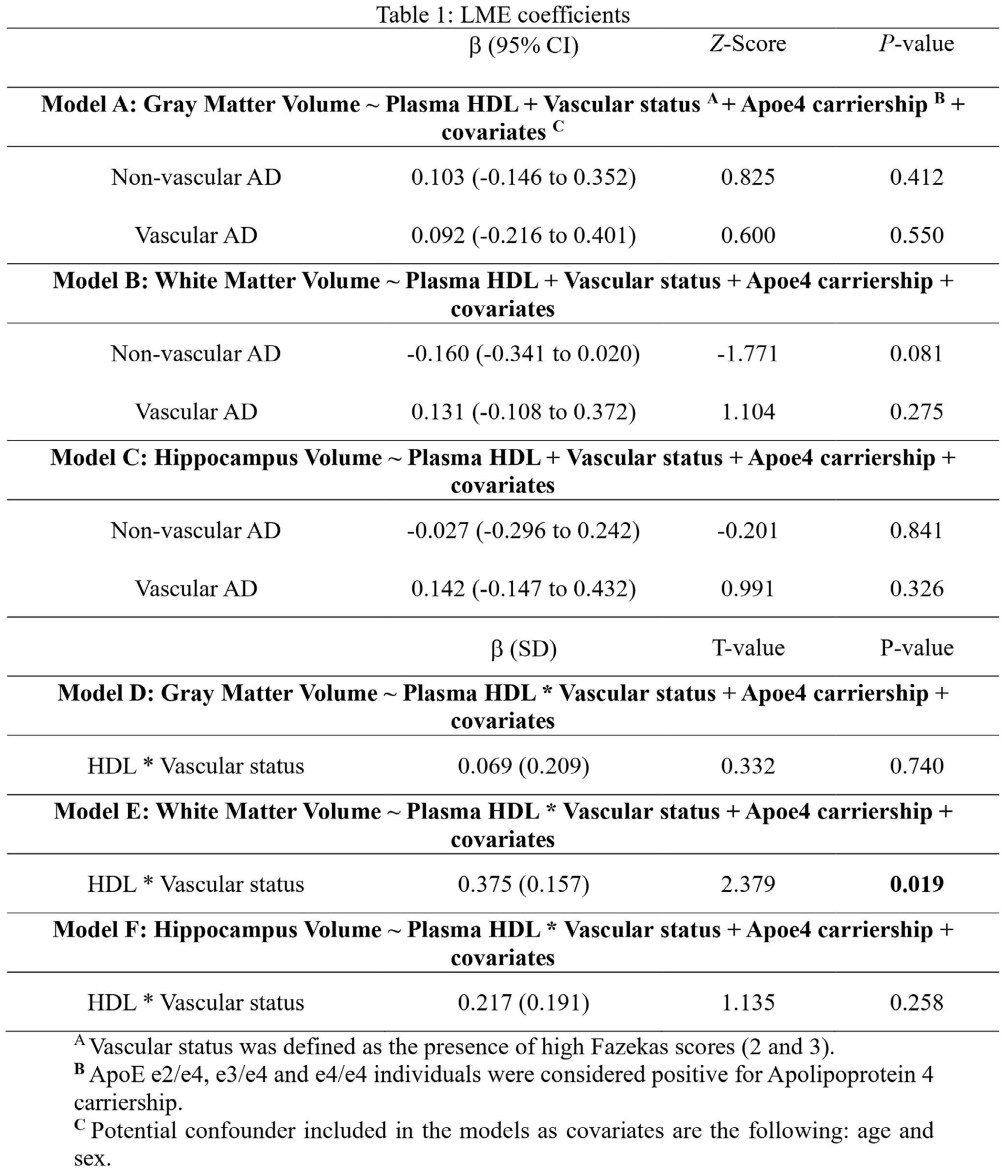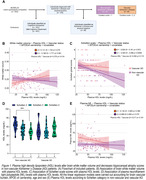# Plasma high‐density lipoprotein levels is associated with preserved hippocampal volume in Alzheimer`s disease individuals without vascular co‐pathology

**DOI:** 10.1002/alz.092693

**Published:** 2025-01-03

**Authors:** Matheus Scarpatto Rodrigues, Markley Silva Oliveira, Firoza Z Lussier, Pamela C.L. Ferreira, Guilherme Povala, Guilherme Bauer‐Negrini, Cynthia Felix, Sarah Abbas, Hussein Zalzale, Carolina Soares, Pampa Saha, Marina Scop Madeiros, Madeleine Bloomquist, Chang‐Hyung Hong, Hyun Woong Roh, Helmet T. Karim, Jade de Oliveira, Thomas K Karikari, Dana Tudorascu, Eduardo R. Zimmer, Bruna Bellaver, Sang Joon Son, Tharick A. Pascoal

**Affiliations:** ^1^ University of Pittsburgh, Pittsburgh, PA USA; ^2^ Department of Psychiatry, University of Pittsburgh School of Medicine, Pittsburgh, PA USA; ^3^ Ajou University School of Medicine, Ajou University Hospital, Suwon, Suwon Korea, Republic of (South); ^4^ Ajou University School of Medicine, Suwon, Gyeonggido Korea, Republic of (South); ^5^ Universidade Federal do Rio Grande do Sul, Porto Alegre Brazil; ^6^ Federal University of Rio Grande do Sul (UFRGS), Porto Alegre, RS Brazil

## Abstract

**Background:**

Dysregulation of cholesterol metabolism contributes to the increase of cerebral vascular diseases, favoring the development of dementia. In this sense, high levels of high‐density lipoprotein (HDL) are considered protective against the outcomes associated with cardiovascular diseases. However, little is known about the effect of plasma HDL levels to alterations of cerebral volume in non‐vascular AD and vascular AD individuals. Here we evaluated the association of plasma HDL levels with alterations in brain volume and with plasma neurofilament light chain (NfL) levels.

**Method:**

We analyzed 135 AD individuals from the Biobank Innovations for Chronic Cerebrovascular Disease with Alzheimer’s Disease Study [BICWALZS] cohort. Based on Fazekas scale for brain white matter lesions, we stratified AD individuals into two groups: non‐vascular AD [Fazekas score = 1 and amyloid‐β (Aβ) positive/n = 76], and vascular AD [Fazekas score = 2, 3 and Aβ positive/n = 59] (**Figure 1A**). Linear regression models accounting for vascular co‐pathology APOEε4 carriership, age and sex were used to test associations of plasma HDL with the variables of interest.

**Result:**

We observed a significant interaction between plasma HDL levels and presence of vascular pathology on total brain white matter (b = 0.375, p = 0.019, **Fig. 1B**; **Table 2**). Analyzing the hippocampal atrophy through the global Schelten`s scale, we observed that high plasma HDL levels was negatively associated with hippocampal atrophy only in non‐vascular AD individuals (b = ‐0.405, p < 0.001, **Fig. 1C**). However, no effect of plasma HDL levels on total gray matter and hippocampal volume was observed (**Table 1**). Non‐vascular AD individuals with high hippocampal atrophy scores showed reduced plasma HDL levels compared to non‐vascular AD individuals with low hippocampal atrophy scores (p < 0.01, **Fig. 1D**). Furthermore, no associations of plasma HDL with NfL levels were detected, regardless of the brain vascular status (**Fig. 1E**).

**Conclusion:**

We showed that high plasma HDL levels is associated with less white matter pathology and reduced hippocampal atrophy scores, without changing plasma NfL levels. Together our findings indicate that high HDL levels seem to be associated with preserved brain volume only in AD individuals with no vascular co‐pathology.